# Estimated Dissemination Ratio—A Practical Alternative to the Reproduction Number for Infectious Diseases

**DOI:** 10.3389/fpubh.2021.675065

**Published:** 2021-07-14

**Authors:** Francisco J. Pérez-Reche, Nick Taylor, Chris McGuigan, Philip Conaglen, Ken J. Forbes, Norval J. C. Strachan, Naomi Honhold

**Affiliations:** ^1^School of Natural and Computing Sciences, University of Aberdeen, Aberdeen, United Kingdom; ^2^Veterinary Epidemiology and Economics Research Unit (VEERU), School of Agriculture, Policy and Development, University of Reading, Reading, United Kingdom; ^3^Department of Public Health and Health Policy, National Health Service (NHS) Lothian, Edinburgh, United Kingdom; ^4^School of Medicine, Medical Sciences and Dentistry, University of Aberdeen, Aberdeen, United Kingdom

**Keywords:** epidemics, survaillance, mathematical models, COVID-19, reproduction number R, estimated dissemination ratio

## Abstract

Policymakers require consistent and accessible tools to monitor the progress of an epidemic and the impact of control measures in real time. One such measure is the Estimated Dissemination Ratio (EDR), a straightforward, easily replicable, and robust measure of the trajectory of an outbreak that has been used for many years in the control of infectious disease in livestock. It is simple to calculate and explain. Its calculation and use are discussed below together with examples from the current COVID-19 outbreak in the UK. These applications illustrate that EDR can demonstrate changes in transmission rate before they may be clear from the epidemic curve. Thus, EDR can provide an early warning that an epidemic is resuming growth, allowing earlier intervention. A conceptual comparison between EDR and the commonly used reproduction number is also provided.

## Key Points

Estimated Dissemination Ratio (EDR) is a simply calculated, replicable, easily explained and robust measure of the trajectory of an outbreak. Examples from the current COVID-19 outbreak in the UK illustrate these merits.

## Introduction

As the severe acute respiratory syndrome coronavirus 2 (SARS-CoV-2) pandemic emerged, policymakers, planners, and frontline workers scrambled to understand the spread and likely impact of this new virus and viral pneumonia it can cause, namely the Coronavirus Disease 2019 (COVID-19). During an epidemic, these public health teams need rapid and reliable information on the progress of the epidemic.

Epidemics have a trajectory and for the planning of responses it is important to understand today what the situation is likely to be tomorrow or next week and in particular, the number of new cases likely to arise. Several different quantitative measures have been used to address these questions. One of the most commonly used is the reproduction number, *R*, which has a long history (1–3). *R* can be described in plain words as “the average number of next-generation cases caused by each current case.” This simple definition refers to a generic *R* which encompasses the basic reproduction number, *R*_0_, when a primary case is introduced to a susceptible population (1, 2, 4) or a reproduction number *R*_t_ determined as a function of time during an epidemic (3, 5). In general, *R* is a measure of the rate of transmission of infection and plays a key role in the management of epidemics. Despite being widely used, however, *R* is itself a complex measure that is difficult to estimate in real time and can easily be misinterpreted by practitioners (6).

An alternative to *R*_*t*_, as an indicator of the rate of transmission of infection during an epidemic, is the Estimated Dissemination Ratio (EDR). At its simplest, EDR is a direct measure of the relative change in the number of cases over time. EDR is a measure that has been used for many years in animal health to monitor progress and control of epidemics, for example with foot and mouth disease (7–10). It has become an established tool for decision support and policy formulation (11–13). As the name implies, EDR can also be interpreted as an estimate of dissemination, or transmission, of infection, since the change in the number of cases over time depends directly on the rate of transmission. EDR gives an estimate of the slope of the epidemic curve and indicates whether an epidemic is accelerating, plateauing—through being brought under control, or declining. It can be an important tool in planning.

Here we discuss the use and value of EDR and provide a conceptual comparison with *R*_*t*_ as a parameter in epidemic management.

## Calculation of EDR

EDR is a simple ratio of cases counted in a set period, divided by the number of cases counted in the preceding period of the same duration. EDR is intentionally simple to calculate using case counts that are available during an epidemic. It can be easily calculated in a transparent, consistent and readily comparable manner. The EDR at day *t* can be calculated by using two consecutive periods of *n* days as follows:

(1) $$\begin{array}{r} EDR=\;\frac{\text{cases in the days }\left[\text{t}-\text{n}+1,\text{t}\right]}{\text{cases in the days }\left[\text{t}-2\text{n}+1,\text{t}-\text{n}\right]} \end{array}$$

For example, for a 7-day period (*n* = 7), EDR is simply given by the cases reported this seven days (between day *t*−6 and day *t*) divided by the cases reported in the previous seven days (between day *t*−13 and day *t*−7).

A 7-day period will be used for illustration in this paper. This choice can be convenient in many applications since it helps to smooth out any anomalous “weekend effects” as seen for example during the current COVID-19 epidemic (14). However, periods other than 7 days could be used and, as discussed below, could be more convenient depending on the specific application.

Since EDR refers to the ratio of cases in two intervals of time, the specific point in time to which EDR is attributed is to some extent arbitrary. Here, we attribute the EDR to the day on which it is calculated, i.e., to the last day in the period used for the numerator in Equation 1. For instance, this is the same convention used to attribute EDR in the freely available “epiR” software package (15). With this choice, a 7 days EDR can be regarded as an indicator of the progression of the epidemic in the last week relative to the week before.

## Interpretation and Use of EDR

The EDR can be interpreted as an approximate indicator of the infection transmission rate. Indeed, assuming that the periods used to calculate EDR approximate the generation interval of the infection, the cases counted in the numerator of EDR (Equation 1) can be considered to be largely generated by contagious transmission from the cases counted in the denominator of EDR. To use EDR as an indicator of infection transmission rate it is important to calculate EDR for time intervals close to the generation time of the disease at hand.

When using EDR to draw inferences about transmission rate, it is important to note that an EDR calculated on current case count data and attributed to the last day in the numerator of Equation 1 will reflect transmission events occurring in the “denominator period” of the EDR–i.e., EDR is a retrospective indicator of transmission rate occurring one generation interval previously.

A graph of EDR over time should be interpreted along with the epidemic curve (case counts or case rates indicating the overall progression and size of the epidemic). Whether there are 2,000 cases in a period following 1,000 cases in a preceding period *or* 20 cases following 10 cases, the EDR equals 2 in both situations. However, disease control decisions might well differ given the different scales. Both the size of the outbreak and the rate at which it is changing (as indicated by EDR) are important. This same consideration applies equally when *R*_*t*_ is used for epidemic management.

An EDR of 1 at a given time indicates that the number of new cases was stable in the preceding periods used to calculate the EDR. An EDR above (or below) 1 indicates that the daily new case numbers increased (or declined) in recent days.

In addition to whether EDR is above or below 1 (cases increasing or decreasing), the absolute value of EDR provides further indication of the speed of increase or decrease of the epidemic curve. When using a 7-day period, an EDR of 2 means that cases are doubling every week, while an EDR of 1.4 means that cases may double in just over 2 weeks. Conversely an EDR of 0.5 means that cases are halving every week; while an EDR of 0.7 means that cases would halve in just under 2 weeks. When EDR is close to 1 (e.g., 0.9–1.1) case numbers are not changing rapidly, but while the situation may not be rapidly deteriorating, neither is it rapidly improving. When EDR is close to 1 it is especially important to also consider the absolute number and spatial distribution of cases. Otherwise, one might miss situations in which the number of cases is maintained at a level from which relaxation of control would result in high case numbers within a relatively short time. The ideal goals in managing an epidemic could be: First reduce the transmission of infection in such a way that the number of new cases fall rapidly (i.e., EDR well-below 1) and, second, maintain this decline until the number of cases is low enough to ensure that individual outbreak clusters can be effectively contained.

Further to looking at the value of EDR at a given time, it is more useful to analyse the trends of EDR. A sustained increase of EDR, when EDR is already >1, indicates an increased transmission rate that will inevitably lead to an acceleration of the increase of cases. More interestingly, an increase of EDR, when EDR is <1, can be observed alongside a decreasing epidemic curve: this indicates that the rate of decline is reducing. This EDR increase would allow us to identify a resurgence of infection which may be difficult to recognize from the epidemic curve. Conversely, an EDR decrease may be observed for an increasing epidemic curve whose rate of increase is reducing, for example, because of interventions implemented to suppress the infection.

## Practical Examples of the Application of EDR in the COVID-19 Outbreak in the Uk

**Figure 1:**
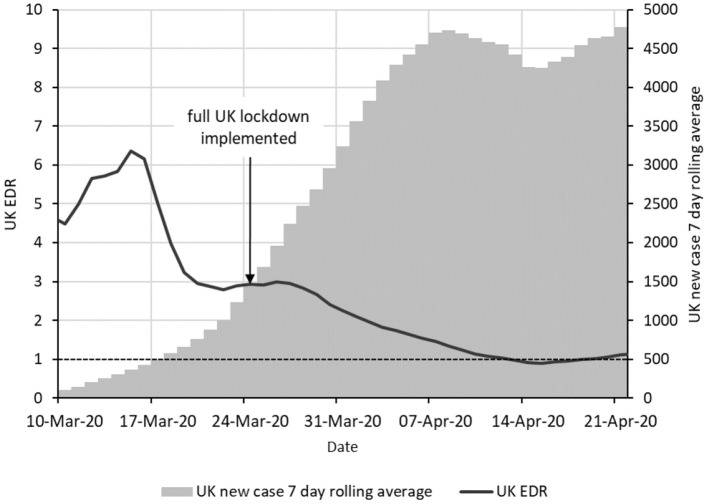
Epidemic curve and EDR for the COVID-19 epidemic in the UK from 1 March to 20 April 2020. The epidemic curve is indicated as vertical bars giving a 7-day moving average of new cases. The solid line shows the EDR estimated using periods of 7 days. The horizontal dashed line shows the boundary with EDR = 1.

Figure 1 shows the epidemic curve and EDR graph for the UK during the COVID-19 epidemic from 10-Mar-20 to 21-Apr-20. EDR was estimated using periods of 7 days (Equation 1). This period is close to the generation interval of COVID-19 (16) and we expect the obtained EDR to be a suitable indicator of the infection transmission rate.

The epidemic curve shows a rapid daily increase in new cases over the period from about 21 March to 1 April which suggests little, if any, control was being achieved. However, the EDR graph shows a sustained and steady downward trend from 18-Mar-20. This suggests a gradual decrease in the transmission of infection over this period. Because EDR is a retrospective indicator of transmission rate, the suggestion is that the transmission rate began falling from around 11 March.

A lockdown was ordered in the UK on 24 March 2020 to suppress the spread of SARS-CoV-2. The declining trend in EDR before this date suggests that transmission was already being slowed before the lockdown, most likely by voluntary home working and reduction in social contact among the population in response to concern over the situation and advice from various sources.

The value of EDR here is that whilst the daily case numbers are increasing quickly, the decreasing EDR shows that there is progress toward control of the epidemic. In this situation, EDR gives an early indication that control measures are working.

**Figure 2:**
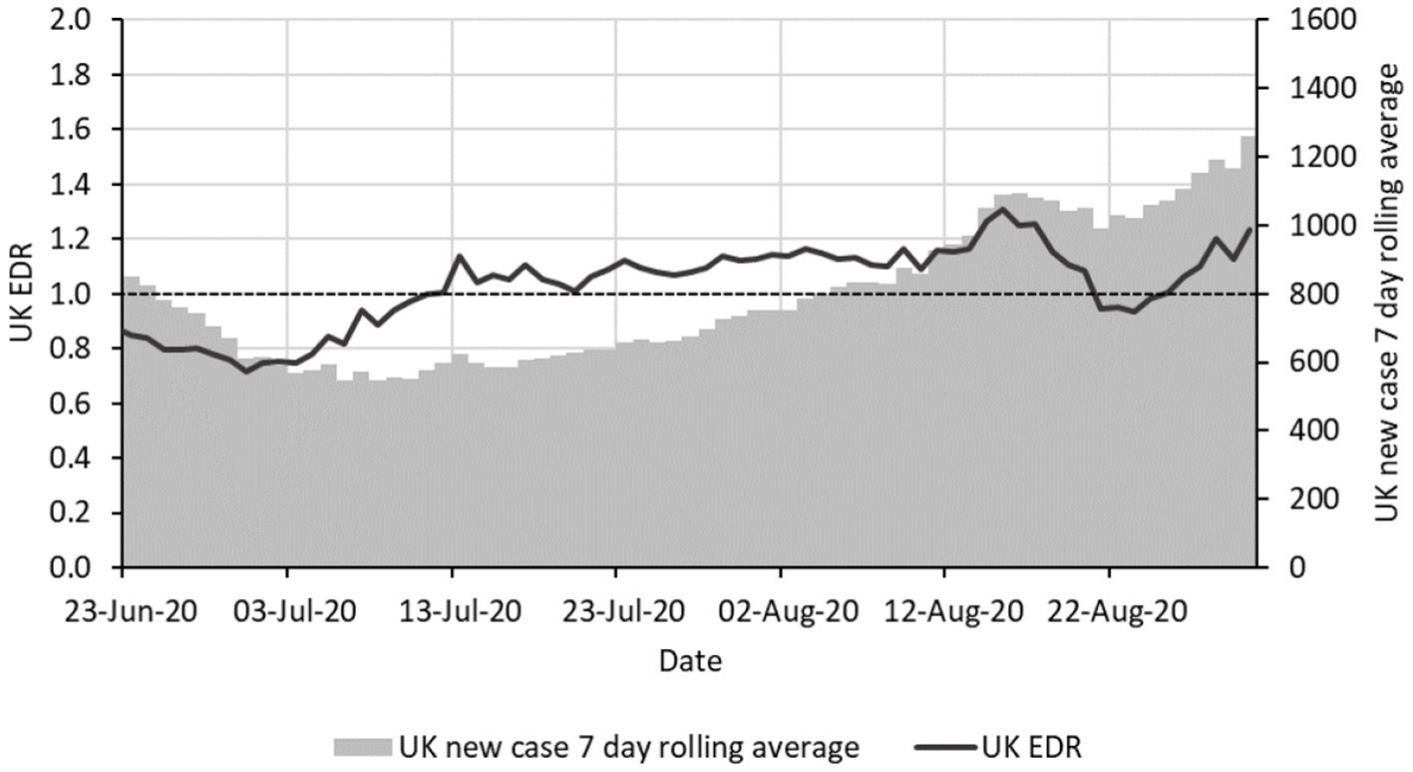
Epidemic curve and EDR for the COVID-19 epidemic in the UK from 23 June to 30 August 2020 with the same format as in Figure 1.

Later in the epidemic, EDR can be used to detect rises in infection rates before they become clear in the case data. This has been more difficult to clearly illustrate because of the changing testing criteria and testing capacity available in the UK which has complicated the picture. Figure 2 show the period from 23-Jun-20 to 30-Aug-20 when testing capacity was stable at between 200,000 and 225,000 tests per day and testing criteria were also stable (17).

EDR started rising consistently from 1 July and was above 1 from 13 July, staying so consistently. The initial increase of EDR suggests a slowing down of the decrease of the epidemic curve before 8 July which gives early signs of a resurge that would indicate a need for action. Despite the signs, no action was taken during this period to prevent a resurge of the virus (18, 19) and cases doubled from around 550 per day on average in early July to around 1,100 by 23 August. As an aside, the EDR fell below 1 on 21 August. It is unclear why this happened as no new interventions were put into place—perhaps a change in testing or reporting, but it can be observed that there was also a slight contemporaneous fall in the new case 7-day moving average.

It would be preferable to use a more dramatic example from a later period of the epidemic but many other conditions have changed since September in the UK including a rapid rise in reported testing capacity and an intensification of testing groups such as school-age children and university students.

Note that in the two examples above, the graphs show EDR and an average daily case number. The latter could equally be replaced with a case rate (for example case per week per 100,000 population) if required.

Especially when using EDR to compare between areas or between time periods, the methods by which cases are defined, searched for, counted and registered must be clearly described. There must be a consistent method of case counting, across the whole period for which comparison of EDR and/or monitoring change is required (*this applies equally to calculations of the reproduction number R*). In practice, this means that changes in either case definition or case searching (surveillance) must be taken into account. Case counts must come from the same population for all times being compared: if surveillance starts to cover a wider population, more cases might be found that are not epidemiologically linked.

## Conceptual Comparison With *R*_t_

*R*_t_ and EDR are similar in several respects. First, both measures indicate the progress of an epidemic and can be used to quantify the infection transmission rate during epidemics. Both EDR and *R*_t_ take values smaller than one for declining epidemics and values larger than one for accelerating epidemics. Apart from the value 1, the two quantities will typically not take identical values for accelerating or declining epidemics.

Despite EDR being qualitatively similar to *R*_t_, the definition and calculation methods of these quantities are significantly different. In principle, an exact estimate of *R*_t_ requires knowing who infected whom during an epidemic. In some cases, it may be possible to construct an epidemic tree to calculate *R*_t_ by simply counting the number of individuals infected by each case (5). For many epidemics, however, it is not known who infected whom and one has to rely on less precise observations such as the epidemic curve. In these situations, estimates of *R*_t_ can be obtained from epidemic curves by fitting mechanistic epidemiological models based on disease-specific assumptions (20–24). Fitting mathematical models to data is often a technically involved task. In addition, models fitted to an epidemic are not easily generalizable to other epidemics. Wallinga and Teunis proposed a more generic method to estimate *R*_t_ which only requires case incidence data and the generation interval distribution (3). This method and some extensions (25, 26) are widely used to estimate *R*_t_. The main drawback of these methods is that they require estimates for the generation interval distribution [or the serial interval as a proxy (3, 25, 26)] which is likely to be missing for emerging diseases. In addition, these methods usually involve advanced mathematical concepts that are not necessarily handled by every public health practitioner. As a consequence, these methods are often perceived as a “black box” of assumptions that are not under the control of practitioners.

In contrast to *R*_t_, EDR only relies on epidemic curves and can be easily estimated without knowledge of advanced mathematical concepts. The period used to calculate EDR can be interpreted as a parameter of the model but its specific value is not absolutely crucial to observe informative trends in EDR. It is interesting, however, that in the particular case in which the period used to calculate EDR approximates the generation time, estimates of *R*_t_ and EDR are expected to be similar to each other.

## Discussion

EDR is intuitive, simple to calculate, easy to explain and relies only on the time series for the number of cases (or other epidemiological observables). As a pragmatic measure of the change in the number of cases over time, EDR can help understand the trajectory of an epidemic in real time. Being unit free, EDR (as with *R*) should always be used in combination with the epidemic curve, allowing both the scale and trajectory of an epidemic to be taken into consideration. The EDR can be a useful indicator of transmission rate as shown here for the COVID-19 outbreak in the UK and previously demonstrated in animal epidemics (7–12). At the very least, EDR is useful as a direct, transparent, measure of the direction (up, down, stable) and the rate of change of the epidemic, but a more in-depth analysis also allows more nuanced interpretation.

EDR is a conservative measure. This is particularly advantageous when EDR is falling. As a retrospective measure, it does not indicate a change until clearly present. Another advantage of displaying EDR along with the daily case totals is that while daily totals can vary considerably from day to day, EDR uses aggregate cases over consecutive multi-day periods, which smooths out the inevitable day-to-day variation.

Being unit free, and given a consistent case definition within a country or territory, EDR can also be used to compare the degree of control between areas. This can be a valuable tool for learning from the experiences of the impact of control measures in different areas.

EDR aims at quantifying the progression of epidemics in a way similar to the reproduction number *R*_t_. Despite some similarities, EDR is significantly easier to estimate than *R*_t_ both in terms of the information required and the mathematical expertise involved. Indeed, a strength of EDR is that it can be readily estimated from epidemic curves. A caveat is that EDR can only capture information of epidemics at the population level. In contrast, *R*_t_ could in principle resolve features of transmission at the level of individuals in cases in which epidemic trees could be reconstructed (5).

## Conclusion

EDR is a transparent measure of the progress of an epidemic with clear potential as a tool to support planning and monitoring of the public health response and impact. It can be used from local to national scales and is readily communicated to the public. The combination of epidemic curve plus EDR is a simple measure of the direction and rate of change in case numbers. This combination can be used as a simple measure to inform epidemic control as well as to explain the progress of outbreaks or to assess the impact of control measures. These multiple uses of EDR together with its clarity should encourage the engagement of frontline workers and the public.

## Data Availability Statement

The original contributions presented in the study are included in the article/supplementary material, further inquiries can be directed to the corresponding author.

## Author Contributions

FP-R, NT, and NH retrieved the data and analyzed it. All authors planned the project, designed the research, wrote the manuscript, reviewed the manuscript, and approved the final version of it.

## Conflict of Interest

The authors declare that the research was conducted in the absence of any commercial or financial relationships that could be construed as a potential conflict of interest.

## References

[1] Anderson RM May RM. Infectious Diseases of Humans: Dynamics and Control. Oxford: Oxford University Press (1991).

[2] Diekmann O Heesterbeek H Britton T. Mathematical Tools for Understanding Infectious Disease Dynamics. Princeton, NJ: Princeton University Press. (2013). 10.1515/9781400845620

[3] Wallinga J Teunis P. Different epidemic curves for severe acute respiratory syndrome reveal similar impacts of control measures. Am J Epidemiol. (2004) 160:509–16. 10.1093/aje/kwh255 15353409 PMC7110200

[4] Heffernan JM Smith RJ Wahl LM. Perspectives on the basic reproductive ratio. J R Soc Interface. (2005) 2:281–93. 10.1098/rsif.2005.0042 16849186 PMC1578275

[5] Haydon DT Chase–Topping M Shaw DJ Matthews L Friar JK Wilesmith J Woolhouse MEJ. The construction and analysis of epidemic trees with reference to the 2001 UK foot–and–mouth outbreak. Proc R Soc Lond Series B: Biol Sci. (2003) 270:121–7. 10.1098/rspb.2002.2191 12590749 PMC1691228

[6] Delamater PL Street EJ Leslie TF Yang YT Jacobsen KH. Complexity of the Basic Reproduction Number (R_0_). Emerg Infect Dis–CDC. (2019) 25:1. 10.3201/eid2501.171901 30560777 PMC6302597

[7] Mansley LM Donaldson AI Thrusfield MV Honhold N. Destructive tension: mathematics versus experience–the progress and control of the 2001 foot and mouth disease epidemic in Great Britain. Rev Sci Tech. (2011) 30:483–98. 10.20506/rst.30.2.2054 21961220

[8] Honhold N Taylor NM Mansley LM Paterson AD. Relationship of speed of slaughter on infected premises and intensity of culling of other premises to the rate of spread of the foot-and-mouth disease epidemic in Great Britain, 2001. Vet Record. (2004) 155:287–94. 10.1136/vr.155.10.287 15478499

[9] Miller W. A state-transition model of epidemic foot and mouth disease. In: Proceedings of an International Symposium: New Techniques in Veterinary epidemiology and Economics. University of Reading (1976). p. 56–72. Available online at: http://www.sciquest.org.nz/elibrary/download/60985/A_state-transition_model_of_epidemic_foot-and-mout.pdf

[10] Thrusfield M Mansley L Dunlop P Pawson A Taylor J. The foot-and-mouth disease epidemic in Dumfries and Galloway, 2001. 2: Serosurveillance, and efficiency and effectiveness of control procedures after the national ban on animal movements. Vet Rec. (2005) 156:269–78. 10.1136/vr.156.9.269 15765895

[11] McCauley EH Aulaqi NA Sundquist WB New JC Miller WM. A study of the potential economic impact of foot and mouth disease in the United States. Proc Annu Meet U S Anim Health Assoc. (1977) 8:284–296. 286348

[12] Morris R Sanson R Stern M Stevenson M Wilesmith J. Decision-support tools for footand- mouth disease control. Revue Scientifique et Technique de l'Office International des Epizooties. (2002) 21:557–67. 10.20506/rst.21.3.1363 12523696

[13] Sanson RL Morris RS Stern MW. EpiMAN-FMD: a decision support system for managing epidemics of vesicular disease. Rev Sci Tech. (1999) 18:593–605. 10.20506/rst.18.3.1181 10588003

[14] Heneghan C. Covid-19 cases and the weekend effect | The Spectator. Available online at: https://www.spectator.co.uk/article/covid-19-cases-and-the-weekend-effect (accessed October 16, 2020)

[15] Stevenson M. epiR: Tools for the Analysis of Epidemiological Data. (2020). Available online at: https://cran.r-project.org/web/packages/epiR/index.html (accessed June 25, 2021)

[16] Flaxman S Mishra S Gandy A Unwin HJT Mellan TA Coupland H. Estimating the effects of non-pharmaceutical interventions on COVID-19 in Europe. Nature. (2020) 584:257–61. 10.1038/s41586-020-2405-7 32512579

[17] Coronavirus (COVID-19) in the UK: Testing. Available online at: https://coronavirus.data.gov.uk/testing (accessed October 27, 2020)

[18] Timeline of the COVID-19 pandemic in the United Kingdom (January–June 2020). Wikipedia. (2020) Available online at: https://en.wikipedia.org/w/index.php?title=Timeline_of_the_COVID-19_pandemic_in_the_United_Kingdom_(January%E2%80%93June_2020)&oldid=985039566 (accessed October 24, 2020)

[19] Timeline of the COVID-19 pandemic in the United Kingdom (July–December 2020). Wikipedia. (2020) Available online at: https://en.wikipedia.org/w/index.php?title=Timeline_of_the_COVID-19_pandemic_in_the_United_Kingdom_(July%E2%80%93December_2020)&oldid=985151520 (accessed October 24, 2020)

[20] Chowell G. Fitting dynamic models to epidemic outbreaks with quantified uncertainty: a primer for parameter uncertainty, identifiability, and forecasts. Infect Dis Mod. (2017) 2:379–98. 10.1016/j.idm.2017.08.001 29250607 PMC5726591

[21] Pérez-Reche FJ Neri FM Taraskin SN Gilligan CA. Prediction of invasion from the early stage of an epidemic. J R Soc Interface. (2012) 9:2085–96. 10.1098/rsif.2012.0130 22513723 PMC3405761

[22] Perez-Reche FJ Forbes KJ Strachan NJC. Importance of untested infectious individuals for the suppression of COVID-19 epidemics. medRxiv. (2020) 0:2020.04.13.20064022. 10.1101/2020.04.13.20064022

[23] Gatto M Bertuzzo E Mari L Miccoli S Carraro L Casagrandi R. Spread and dynamics of the COVID-19 epidemic in Italy: effects of emergency containment measures. PNAS. (2020) 117:10484–91. 10.1073/pnas.2004978117 32327608 PMC7229754

[24] López L Rodó X. The end of social confinement and COVID-19 re-emergence risk. Nat Hum Behav. (2020) 584:257–261. 10.1101/2020.04.14.20064766 32572175

[25] Cori A Ferguson NM Fraser C Cauchemez S A. New framework and software to estimate time-varying reproduction numbers during epidemics. Am J Epidemiol. (2013) 178:1505–12. 10.1093/aje/kwt133 24043437 PMC3816335

[26] Thompson RN Stockwin JE van Gaalen RD Polonsky JA Kamvar ZN Demarsh PA. Improved inference of time-varying reproduction numbers during infectious disease outbreaks. Epidemics. (2019) 29:100356. 10.1016/j.epidem.2019.100356 31624039 PMC7105007

